# Mapping the Fat: How Childhood Obesity and Body Composition Shape Obstructive Sleep Apnoea

**DOI:** 10.3390/children12070912

**Published:** 2025-07-10

**Authors:** Marco Zaffanello, Angelo Pietrobelli, Giorgio Piacentini, Thomas Zoller, Luana Nosetti, Alessandra Guzzo, Franco Antoniazzi

**Affiliations:** 1 Department of Surgical Sciences, Dentistry, Gynecology and Pediatrics, University of Verona, 37129 Verona, Italy; angelo.pietrobelli@univr.it (A.P.); giorgio.piacentini@univr.it (G.P.); thomas.zoller@univr.it (T.Z.); franco.antoniazzi@univr.it (F.A.); 2 Department of Medicine and Technological Innovation, Insubria University, F del Ponte Hospital, 21100 Varese, Italy; luana.nosetti@uninsubria.it; 3 Laboratory Unit, Department of Neurosciences, Biomedicine and Movement Sciences, University of Verona, 37129 Verona, Italy

**Keywords:** anthropometric measure, body composition, children, fat distribution, obesity, obstructive sleep apnoea, polysomnography, sleep disordered breathing, ultrasonography

## Abstract

**Background/Objectives**: Childhood obesity represents a growing public health concern. It is closely associated with obstructive sleep apnoea (OSA), which impairs nocturnal breathing and significantly affects neurocognitive and cardiovascular health. This review aims to analyse differences in fat distribution, anthropometric parameters, and instrumental assessments of paediatric OSA compared to adult OSA to improve the diagnostic characterisation of obese children. **Methods**: narrative review. **Results**: While adenotonsillar hypertrophy (ATH) remains a primary cause of paediatric OSA, the increasing prevalence of obesity has introduced distinct pathophysiological mechanisms, including fat accumulation around the pharynx, reduced respiratory muscle tone, and systemic inflammation. Children exhibit different fat distribution patterns compared to adults, with a greater proportion of subcutaneous fat relative to visceral fat. Nevertheless, cervical and abdominal adiposity are crucial in increasing upper airway collapsibility. Recent evidence highlights the predictive value of anthropometric and body composition indicators such as neck circumference (NC), neck-to-height ratio (NHR), neck-to-waist ratio (NWR), fat-to-muscle ratio (FMR), and the neck-to-abdominal-fat percentage ratio (NAF%). In addition, ultrasound assessment of lateral pharyngeal wall (LPW) thickness and abdominal fat distribution provides clinically relevant information regarding anatomical contributions to OSA severity. Among imaging modalities, dual-energy X-ray absorptiometry (DXA), bioelectrical impedance analysis (BIA), and air displacement plethysmography (ADP) have proven valuable tools for evaluating body fat distribution. **Conclusions**: Despite advances in the topic, a validated predictive model that integrates these parameters is still lacking in clinical practice. Polysomnography (PSG) remains the gold standard for diagnosis; however, its limited accessibility underscores the need for complementary tools to prioritise the identification of children at high risk. A multimodal approach integrating clinical, anthropometric, and imaging data could support the early identification and personalised management of paediatric OSA in obesity.

## 1. Introduction

### 1.1. Paediatric Obesity

Childhood obesity has emerged as a significant public health concern, with serious long-term implications [1]. Its prevalence has risen markedly in recent decades, primarily attributable to sedentary lifestyles, unhealthy dietary habits, and genetic predispositions [1,2,3]. Evidence suggests that primary school age (6–11 years) and adolescence constitute critical developmental windows during which environmental factors, hormonal fluctuations, and behavioural changes significantly increase the risk of obesity onset [4,5,6,7,8]. This condition is associated with a significantly elevated risk of multiple complications, including insulin resistance, type 2 diabetes mellitus [1,9], hypertension, dyslipidaemia, cardiovascular diseases, and a range of psychological and social challenges, as low self-esteem, depression, and social stigma, thereby perpetuating and exacerbating existing health inequalities [1,9,10,11].

Metabolic syndrome is a cluster of interconnected conditions including central obesity, insulin resistance, hypertension, and dyslipidaemia, which collectively increase the risk of cardiovascular disease. It frequently coexists with paediatric obesity and significantly contributes to the development and severity of obstructive sleep apnoea (OSA). Globally, the prevalence of obesity in children has risen dramatically in recent decades, with current estimates indicating that over 340 million children and adolescents aged 5–19 years were overweight or obese in 2016 (WHO) [12].

### 1.2. Systemic Inflammation and Neuromuscular Control in Paediatric OSA

OSA is a sleep-related breathing disorder characterised by recurrent episodes of partial or complete upper airway obstruction during sleep, leading to intermittent hypoxia, sleep fragmentation, and impaired gas exchange [13]. In children, OSA can result in neurocognitive impairment, behavioural disturbances, growth impairment, and cardiovascular complications. OSA is closely linked to childhood obesity [14,15].

Low-grade systemic inflammation plays a pivotal role in the pathophysiology of obesity-related paediatric OSA [16,17]. This process is associated with immune cell infiltration into adipose tissue and increased levels of circulating pro-inflammatory cytokines. As detailed in the subsequent sections, these changes contribute to airway instability during sleep through multiple mechanisms.

Simultaneously, adipose tissue accumulation in the pharyngeal structures undermines airway stability during sleep. In obese adults, chronic inflammation associated with obesity impairs the compensatory function of the pharyngeal dilator muscles, which is crucial for maintaining airway patency [18,19]. Dysfunction of key muscles, such as the genioglossus, further heightens susceptibility to airway collapse and obstructive sleep events [20].

### 1.3. Sleep-Disordered Breathing

#### 1.3.1. Pathophysiological Links Between Paediatric Obesity and OSA

Low-grade systemic inflammation is a major contributor to the pathophysiology of obesity-related paediatric OSA [16,17]. From a histological perspective, it is typified by the infiltration of macrophages and lymphocytes into adipose tissue [21], along with elevated circulating levels of pro-inflammatory cytokines such as TNF-α and IL-6 [22], which collectively promote oxidative stress and endothelial dysfunction within the airways [23,24]. This chronic inflammatory environment induces remodelling of the upper airway architecture, including submucosal thickening, increased collagen deposition, and reduced tissue elasticity [25], thereby increasing susceptibility to airway collapse during sleep [26]. Furthermore, inflammatory mediators may potentiate bronchial hyperresponsiveness, further heightening the risk of airway compromise.

Simultaneously, fat deposition within the pharyngeal structures undermines the mechanical stability of the airway during sleep. In obese adults, upper airway neuromuscular control is also compromised; the compensatory function of the pharyngeal dilator muscles, essential for maintaining airway patency, is impaired by obesity-related chronic inflammation [18,19]. Dysfunction of critical muscles such as the genioglossus increases the risk of airway collapse and obstructive events [27].

#### 1.3.2. Differences in Pathogenesis of Sleep-Disordered Breathing in Obese Adults and Children

Sleep-disordered breathing (SDB), including OSA, occurs more frequently in obese adults than in obese children [28,29]. Among adults with severe obesity (body mass index, BMI > 40 kg/m^2^), the prevalence of OSA ranges between 40% and 90% [30,31], being up to four times more prevalent in males and up to seven times more frequent in those with BMI ≥ 30 [32,33].

In the general paediatric population, OSA prevalence is estimated at 1–5% [34], but among obese children, it increases substantially, with rates ranging from 13% to 60% [35,36]. Despite this marked increase, the prevalence of OSA in paediatric populations remains lower than that observed in obese adults [37].

In adults, greater pharyngeal and cervical fat deposition and age-related muscular degeneration increase susceptibility to OSA [38,39,40]. While SDB is less prevalent in children, it remains clinically significant, particularly in the presence of predisposing factors such as adenotonsillar hypertrophy (ATH) [41].

Compared to adults, obese children demonstrate greater neuromuscular adaptability and distinct anatomical features of the upper airway, which may reduce the likelihood of complete airway obstruction [42]. It is essential to note that the pathophysiological mechanisms underlying paediatric obesity differ significantly from those in adults.

#### 1.3.3. Comorbid Adenotonsillar Hypertrophy in Obesity and OSA

Obesity constitutes a significant risk factor for SDB; however, its impact differs between adults and children [43]. Obese children show a higher prevalence of SDB compared to their normal-weight peers, with estimates suggesting that up to 60% are affected [28,44]. In children, the risk of OSA increases significantly when obesity coexists with ATH [43,45].

ATH is the primary cause of paediatric OSA, peaking between 2 and 6 years of age [46]. ATH may significantly obstruct the upper airway during sleep, resulting in intermittent apnoeic episodes, oxygen desaturation, and sleep fragmentation [47]. In obese children, airway obstruction is worsened by pharyngeal fat accumulation and impaired neuromuscular control of the upper airway [48].

Metabolic syndrome, frequently observed in obese children, is characterised by insulin resistance, hypertension, dyslipidaemia, and visceral adiposity [49]. This syndrome contributes to OSA onset and severity through fat accumulation in the abdominal and cervical regions [50,51] and reducing airway muscle tone due to inflammation and hormonal disturbances [52]. The coexistence of obesity, metabolic syndrome, and OSA substantially increases long-term cardiovascular and metabolic risk [50].

Moreover, both obesity and SDB are frequently observed in genetic syndromes, such as Down syndrome and Prader–Willi syndrome. In Down syndrome, hypotonia and craniofacial abnormalities (macroglossia, short neck) increase obstruction risk [53,54], whereas in Prader–Willi syndrome, hypothalamic dysfunction leads to severe early-onset obesity and a higher risk of SDB [55,56].

When paediatric obesity coexists with ATH, the risk of upper airway obstruction rises considerably [57,58]. In obese adults, pharyngeal fat may further worsen the obstruction caused by enlarged tonsils and adenoids [59], leading to more severe episodes of OSA, greater sleep fragmentation, and more pronounced nocturnal oxygen desaturations [47,60]. Chronic low-grade inflammation in obesity further increases upper airway resistance, establishing a vicious cycle that progressively worsens respiratory function [61,62].

The diagnosis of OSA in obese children requires a thorough clinical assessment. Polysomnography (PSG) remains the gold standard [28], although tools like the Paediatric Sleep Questionnaire and Epworth Sleepiness Scale can help identify high-risk individuals [28,41]. Assessment of anthropometric and metabolic indicators—BMI, neck circumference (NC), and specific biomarkers—is also essential for risk stratification [29].

Effective management of OSA in obese children requires an integrated approach that addresses anatomical, metabolic, and inflammatory components simultaneously.

#### 1.3.4. Methods

This study is a narrative review based on a structured, though non-systematic, literature search. Relevant articles were identified through PubMed, Scopus, and Web of Science, using combinations of keywords such as “paediatric obstructive sleep apnea”, “childhood obesity”, “body composition”, “anthropometric parameters”, and “sleep-disordered breathing”. The search was limited to English-language publications up to March 2025. Additional references were retrieved from the bibliographies of selected articles. The key search terms included “paediatric obstructive sleep apnea”, “childhood obesity”, “body composition”, “anthropometric parameters”, and “sleep-disordered breathing”. We included clinical studies, imaging studies, and reviews focusing on paediatric populations, while excluding case reports, editorials, and non-English articles. As this is a narrative review, no formal quality assessment of the included studies was performed.

As this is a narrative review, no formal quality assessment of the included studies was performed. To highlight unique aspects of childhood obesity-related OSA, adult studies were included exclusively to provide comparative insights regarding anatomical, physiological, and pathophysiological differences between paediatric and adult OSA.

## 2. Aims

This study aims to provide a critical appraisal of the literature on differences in body fat distribution, anthropometric measures, and instrumental evaluation of OSA in obese children as compared to obese adults. The specific aims are as follows: (1) to delineate the variations in body fat distribution between paediatric and adult populations with obesity; (2) to appraise the diagnostic relevance of anthropometric parameters in children; and (3) to investigate the utility of paediatric-adapted instrumental assessments in the characterisation and clinical management of OSA.

## 3. Body Fat Distribution in Obese Children

### 3.1. Differences in Body Fat Distribution Between Obese Prepubertal Children and Adults

Body fat distribution in obese prepubertal children differs markedly from that in obese adults. In children, obesity is typically characterised by a predominance of subcutaneous fat [63,64], whereas in adults, adiposity is primarily concentrated in visceral and abdominal regions [65]. This distinction is clinically important, as visceral fat is metabolically active and strongly linked to insulin resistance, type 2 diabetes mellitus, and cardiovascular disease [65,66].

However, children with severe obesity may show early visceral fat accumulation, predisposing them to long-term metabolic complications [67,68]. Additionally, fat deposition in the thoracic and cervical areas of obese children may contribute to the development of OSA [69,70], although both the prevalence and severity of the condition are generally lower than in adults.

### 3.2. Gender Differences in Body Fat Distribution Among Prepubertal Children

Patterns of body fat distribution differ substantially between prepubertal boys and girls [71]. Boys tend to exhibit a more uniform distribution, with a mild predominance of abdominal fat, while girls demonstrate greater subcutaneous fat accumulation in the gluteal and femoral regions [72]. These sex-specific differences become more pronounced at puberty. Rising oestrogen levels in girls promote fat storage in the gluteofemoral region [73], whereas increasing testosterone levels in boys enhance muscle development and reduce overall body fat in healthy individuals [74].

### 3.3. Hormonal Influences on Body Fat Distribution from Childhood Through Adolescence and Adulthood

Significant changes in body fat distribution occur during childhood to adulthood, driven primarily by hormonal changes [75,76]. Pubertal increases in sex hormones—especially oestrogen and testosterone—promote redistribution of adipose tissue [75,77]. Additional metabolic hormones, including insulin, cortisol, and leptin, also play key roles in lipid metabolism and fat distribution [78,79,80]. Insulin regulates both lipogenesis and lipolysis; in obese children, insulin resistance disrupts these processes, favouring visceral fat accumulation and reducing lean mass [81].

Early onset of secondary sexual characteristics is associated with increased cardiovascular risk in paediatric populations, particularly in cases of precocious pubarche [82]. This condition is often linked to hyperinsulinaemia, which may contribute to central adiposity and the early development of metabolic syndrome [83,84]. These endocrine and metabolic alterations should be considered in the risk stratification of obese children, as they may exacerbate cardiometabolic burden and influence OSA pathophysiology [85].

### 3.4. Upper Airway Fat Distribution in Paediatric OSA

Paediatric OSA severity is influenced by BMI percentile and age [86].

Fat distribution in the upper airway is a critical factor in the pathophysiology of OSA [58,87].

Imaging studies have shown that patients with OSA have greater adipose tissue volumes around the pharyngeal airway than BMI-matched controls [38,88].

Lateral pharyngeal fat pads exert inward pressure on the pharyngeal walls, increasing airway collapsibility, strongly correlating with the apnoea–hypopnoea index (AHI) [89].

Weight loss significantly reduces pharyngeal fat volume, leading to a decrease in apnoeic events [88].

MRI studies confirm that increased lateral pharyngeal wall (LPW) soft tissue volume compresses the airway externally, increasing OSA risk [90].

Excessive anterolateral LPW fat has been found even in non-obese individuals with OSA compared to controls [91].

Axial CT imaging of the oropharynx shows that parapharyngeal fat pads significantly contribute to airway crowding and upper airway collapse 92,89 .

Patients with OSA have significantly enlarged tongues with increased intramuscular adipose tissue specially in the posterior third [93].

In both adults [94] and children [95], fat-enlarged tongues are more prone to posterior displacement in the supine position, contributing to increased pharyngeal critical closing pressure (Pcrit) and airway collapse [96].

## 4. Anthropometric Parameters

### 4.1. Neck Circumference and Neck-to-Height Ratio

NC is a simple, non-invasive, reproducible measurement that is an effective screening tool for identifying children at increased risk of OSA.

Assessment of NC is considered important in the clinical evaluation of OSA in children and adults [97].

In a study involving 507 paediatric participants (62% male) aged 5 to 18, the authors proposed a predictive model for OSA in children based on the neck-to-height ratio (NHR) and BMI z-score (zBMI).

The final model included NHR, zBMI, and the interaction term NHR × zBMI.

Neither NHR nor zBMI alone was a reliable predictor; however, the combination of elevated NHR and increased zBMI strongly predicted OSA risk.

The interaction between NHR and zBMI was the most significant predictor of the AHI.

Another study confirmed that an increase in NC is a reliable indicator of OSA risk, as it contributes to upper airway narrowing in obese children [98].

In this study, which included 71 children (median age: 14.8 years; interquartile range [IQR]: 12.6–16.0; 54% male), a 0.01-unit increase in NHR was associated with a 55% increase in the obstructive AHI (oAHI) (OR: 1.55; 95% CI: 1.36–1.80; *p* < 0.001).

These findings support the use of the NHR, rather than NC alone, as a more accurate predictive marker of OSA in obese children.

Moreover, the model suggested a synergistic interaction between an elevated NHR and male sex in predicting increased oAHI (≥5 events/hour) [98].

### 4.2. Neck Circumference, Waist Circumference, and Neck-to-Waist Ratio

Sukharom et al. examined 132 obese children (76.5% male; mean age: 12.5 ± 3.2 years) [99]. The authors found that a BMI greater than 29.2 kg/m^2^ was significantly associated with severe OSA, with a sensitivity of 81.3% and a specificity of 48.5% (OR: 4.08; 95% CI: 1.85–9.00). An NC exceeding 35.8 cm was also identified as a significant predictor of severe OSA, yielding a sensitivity of 84.4% and a specificity of 47.0% (OR: 4.78; 95% CI: 2.09–10.97). Similarly, a waist circumference (WC) ≥ 93.5 cm was associated with severe OSA (sensitivity: 82.8%; specificity: 34.8%; OR: 2.58; 95% CI: 1.13–5.87).

**Figure 1:**
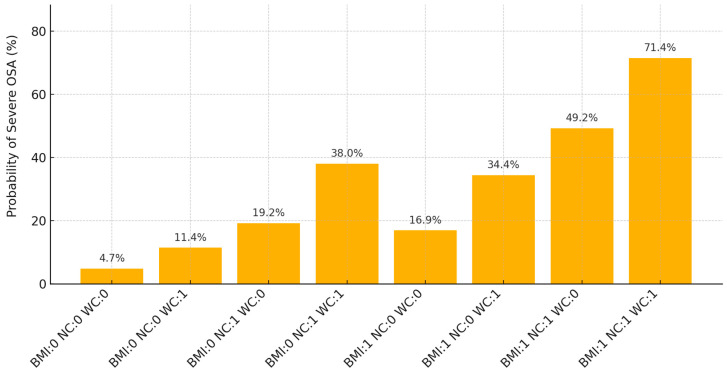
Estimated probability of severe paediatric OSA (AHI ≥ 10 events·h^−1^) derived from multivariable logistic regression according to combinations of BMI > 29.2 kg·m^−2^, neck circumference > 35.8 cm, and waist circumference ≥ 93.5 cm (n = 152). Bars represent predicted probability (%) with 95% confidence intervals; dashed horizontal lines mark 25%, 50%, and 75% probability thresholds. Abbreviations: BMI, body mass index; NC, neck circumference; WC, waist circumference.

The estimated probabilities (%) of severe OSA based on various combinations of BMI, NC, and WC thresholds in paediatric subjects are illustrated in Figure 1.

However, risk assessment in obese children should incorporate specific indicators of body fat distribution, such as the neck-to-waist or neck-to-abdomen ratio, in addition to standard clinical criteria. Katz et al. evaluated 222 children, of whom 133 (60%) were overweight or obese and 121 (55%) were male; 47 children (21%) were diagnosed with OSA. The median age of the study population was 12.1 years (range: 7.0–17.9 years).

The NWR and zBMI were identified as independent, statistically significant predictors of OSA [100].

### 4.3. Waist-to-Hip Ratio

In a systematic review, De Araújo Lopes et al. assessed the utility of the waist-to-hip ratio (WHR) in the diagnosis and risk stratification of OSA among obese children and adolescents [101].

NC was identified as the most reliable anthropometric marker, supported by a high level of evidence for its association with paediatric OSA.

**Figure 2:**
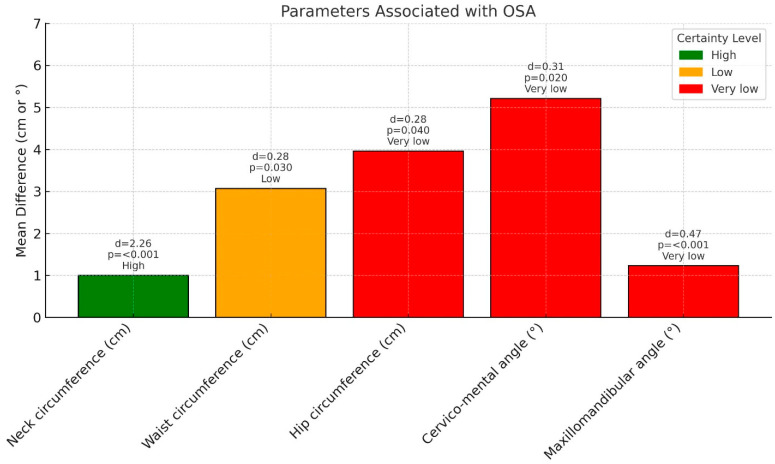
Standardised mean differences (Cohen’s d) in anthropometric and craniofacial parameters between children with and without obstructive sleep apnoea (OSA). The bars show differences in neck, waist, and hip circumferences (cm), and cervicomental and maxillomandibular angles (degrees). Bar colours indicate certainty of evidence: green = high, orange = low, red = very low [101]. Abbreviations: NC, neck circumference; WC, waist circumference; WHR, waist-to-hip ratio.

Figure 2 presents a bar chart illustrating the mean differences in selected parameters between children with and without OSA, with each bar colour-coded according to the certainty of evidence (green = high, orange = low, red = very low).

Labels above each bar indicate the corresponding effect size (Cohen’s *d*), *p*-value, and level of certainty. The parameters considered include neck, waist, and hip circumferences (in centimetres) and cervicomental and maxillomandibular angles (measured in degrees). Statistical significance and effect size were used to quantify and compare the magnitude of differences between groups.

A study confirmed that an elevated WHR is a reliable marker of visceral obesity in healthy adults [102].

Among men with a mean age of 52 ± 9 years, a higher WHR was associated with greater severity of OSA [103]. However, the relationship between WHR and SDB risk in children has not been extensively investigated. Central obesity in children—often assessed using the waist-to-height ratio [104] and WHR [105]—has been more consistently associated with OSA risk than WHR alone.

Available evidence suggests that WC [101] and the NWR [98] are more robust predictors of OSA in children than WHR, although some studies have indicated an indirect association [101,106].

Notably, a study conducted in prepubertal children found no significant differences in the WHR between those with SDB and healthy controls [107].

## 5. Instrumental Evaluation

### 5.1. Ultrasound

#### 5.1.1. Neck Ultrasonography

Ultrasound (US) is a non-invasive and valuable technique for monitoring anatomical changes following therapeutic interventions, such as weight loss or adenotonsillectomy.

A recent systematic review highlighted its utility in evaluating upper airway anatomy and identifying potential predisposing abnormalities in adults and children with OSA [108].

Neck US may have significant clinical value in the assessment of paediatric OSA. The LPW thickness, measured sonographically both at rest and during the Müller manoeuvre, showed a moderate but significant correlation with OSA severity (as measured by the AHI), and may represent an independent structural risk factor in children (82 participants; mean age: 7.7 ± 6.2 years; 76% male) [109].

An earlier study confirmed the intra- and inter-operator reliability of LPW thickness measurements in a cohort of 34 children (mean age: 8.66 ± 1.61 years; 26 males) across varying degrees of OSA severity, thereby providing a robust evaluation of the association between LPW thickness and disease severity [110].

Although US provides valuable anatomical insights during the initial assessment, it does not replace PSG, which remains the gold standard for the definitive diagnosis of OSA [111].

#### 5.1.2. Ultrasound Assessment of Abdominal Fat

US evaluation of abdominal adiposity provides important information regarding the relationship between obesity and OSA in children [69]. Research has demonstrated that visceral adiposity negatively affects pulmonary mechanics and increases upper airway resistance in paediatric patients [112]. Compared to subcutaneous fat, visceral fat accumulation is more strongly associated with impaired respiratory function, particularly among obese females [113].

In adults, abdominal adiposity has been shown to exert mechanical pressure on the diaphragm, leading to reduced functional residual capacity and an increased risk of nocturnal hypoventilation [114,115]. US imaging enables a direct, non-invasive assessment of visceral adipose tissue [116,117], allowing clinicians to correlate the degree of obesity with OSA severity.

Moreover, serial US assessments can be employed to monitor the effectiveness of interventions targeting visceral fat reduction [118], thereby supporting personalised therapeutic strategies for obese children with OSA.

#### 5.1.3. Polysomnography and Neck Ultrasonography

PSG provides a detailed functional assessment of nocturnal breathing, quantifying apnoeic episodes, oxygen desaturations, and sleep fragmentation.

However, US offers a non-invasive means of evaluating anatomical parameters, such as tongue base and LPW thickness.

When used together, PSG and US represent complementary diagnostic tools for assessing OSA, particularly in adults [119].

Point-of-care US has also emerged as a potential screening method for OSA, enabling the assessment of multiple anatomical and physiological parameters—including tongue volume, tonsil size, pharyngeal dimensions, and the abdominal fat index [120].

Neck US has demonstrated clinical utility in assessing 100 patients aged 18 to 70, allowing for measurement of LPW thickness, tonsillar volume, and the distance between the tongue base and the posterior pharyngeal wall in obese adults [121,122,123].

These parameters are associated with an increased risk of OSA and may also be relevant in the paediatric population.

Furthermore, US allows for real-time visualisation of respiratory dynamics and upper airway collapse in cadaveric models under simulated sleep conditions [124].

Recent studies have revealed morphological and functional differences in upper airway structures among patients with OSA [108].

Integrating PSG with neck US could enhance diagnostic accuracy and inform more targeted therapeutic strategies, such as adenotonsillectomy.

While PSG remains indispensable for the functional evaluation of SDB, US provides valuable anatomical insights, particularly regarding LPW thickness.

A study demonstrated a statistically significant increase in LPW thickness (*p* < 0.001) in patients with OSA compared to controls [125].

In a study of 82 obese children (mean age: 7.7 ± 6.2 years) with suspected OSA, the mean total thickness of the LPW and neck at the retropalatal level was significantly greater in children with OSA than in those with primary snoring [109].

Moreover, submental US assessment of the retroglossal (RG) and retropalatal (RP) regions has proven capable of accurately discriminating OSA severity in adults (105 participants) [126].

These two modalities (PSG and US) may facilitate clinical decision-making regarding the most appropriate therapeutic interventions, including adenotonsillectomy or nutritional management.

US is also valuable in the follow-up setting for monitoring outcomes after conservative or surgical treatments in adults.

Backscatter US tongue imaging has been correlated with OSA severity [127].

Adult patients with obstructive sleep apnoea–hypopnoea syndrome (OSAHS) (n = 104) exhibited narrower airway diameters, more circular airway shapes, and greater dynamic changes in oropharyngeal dimensions during deep respiration compared to healthy controls [128].

Thus, US can identify dimensional changes in the oropharyngeal airway that reflect disease severity and response to treatment.

### 5.2. Body Composition

#### 5.2.1. Body Composition Assessment Is Essential in the Clinical Evaluation of Paediatric Obesity

Reliable techniques include skinfold thickness measurement, air displacement plethysmography (ADP), dual-energy X-ray absorptiometry (DXA), and US [129,130].

Methods such as bioelectrical impedance analysis (BIA) [131,132], DXA [133,134], and ADP [135,136,137] allow for accurate differentiation between FM and LM.

In a study involving 74 children aged 2 to 6, body fat percentage was assessed using ADP and compared with values obtained from the four-compartment model.

#### 5.2.2. Body Composition Analysis in Managing Obese Children with OSA

**Figure 3:**
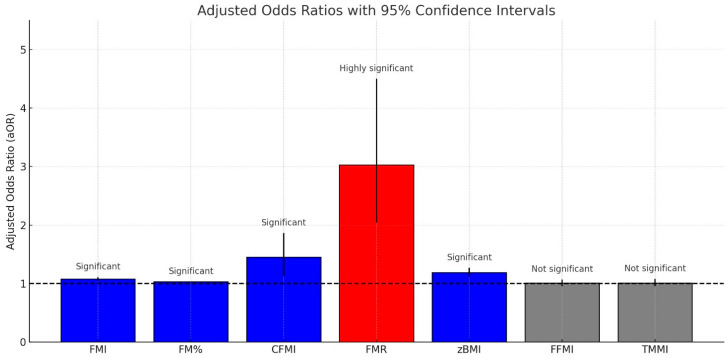
Adjusted odds ratios (aORs) and 95% confidence intervals (CIs) for various body composition indicators and their association with paediatric obstructive sleep apnoea (OSA). Indicators: CFMI = central fat mass index; FFMI = fat-free mass index; FMI = fat mass index; FMR = fat-to-muscle mass ratio. Bar colour coding: red = highly significant (*p* < 0.001); blue = significant (*p* < 0.05); grey = non-significant (*p* > 0.05). The dashed horizontal line at aOR = 1.0 indicates the null association [45].

Body composition analysis plays a key role in the management of obese children diagnosed with OSA. A significant association has been reported between body composition parameters and the risk of OSA (Figure 3) [45].

#### 5.2.3. Fat-to-Muscle Ratio and Risk of OSA

Among all indicators, the fat-to-muscle ratio (FMR) demonstrated the strongest association with OSA risk [45].

Total FM, central FM, and FMR were independently associated with the risk of OSA in children and adolescents, irrespective of lean mass indicators.

In a separate study, Glicksman et al. proposed a predictive formula linking body composition to OSA risk in obese youth [138].

The neck-to-abdominal fat percentage (NAF%) ratio was identified as a more sensitive predictor of OSA risk than BMI alone.

A higher proportion of cervical fat relative to abdominal fat was significantly associated with an increased risk of OSA (*p* = 0.03).

Detailed assessment of FM and its distribution provides critical insights into the risk and severity of upper airway obstruction.

Increased adiposity in the cervical and pharyngeal regions is associated with greater upper airway collapsibility, a key factor in the pathogenesis of OSA [18,91].

Furthermore, body composition analysis is valuable for evaluating treatment responses following interventions such as weight loss programmes and lifestyle modifications, supporting a more targeted and individualised therapeutic approach.

#### 5.2.4. Polysomnography and Body Composition Analysis in Paediatric OSA Management

According to findings from a study by Glicksman et al., in which both PSG and DXA were performed, neither FM nor FFM demonstrated a significant correlation with severe OSA in obese children and adolescents. The study included 30 obese participants with a median age of 14.5 years (interquartile range [IQR]: 11.5–15.5; overall range: 7.8–17.9 years) [138].

**Figure 4:**
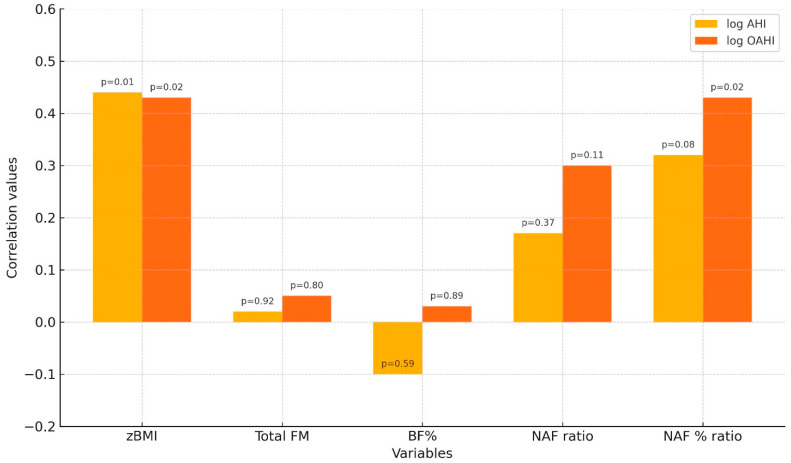
Pearson correlation coefficients between the log-transformed apnoea–hypopnoea index (log AHI)/obstructive apnoea–hypopnoea index (log oAHI) and adiposity indicators. Variables: BMI z-score (zBMI), total fat mass (FM), body fat percentage (BF%), neck-to-abdominal fat percentage ratio (NAF%). Bars display correlation coefficients, with p-values shown above each bar. Significant associations are indicated [138].

The bar chart (Figure 4) illustrates correlation values (r) between log AHI and log oAHI and five adiposity-related variables—zBMI, total fat mass (Total FM), body fat percentage (BF%), the ratio of neck fat mass to abdominal fat mass (NAF ratio), and the neck-to-abdominal fat percentage (NAF % ratio) ratio. Corresponding p-values are displayed above each bar. Significant correlations were observed between log AHI and zBMI (r = 0.44, *p* = 0.01) and between log oAHI and both zBMI (r = 0.43, *p* = 0.02) and the NAF% ratio (r = 0.43, *p* = 0.02) [138].

The NAF% were significantly correlated with the log-transformed obstructive AHI (log oAHI) (*p* = 0.02 for both).

In contrast, FM and BF% were not independently associated with log oAHI.

Only markedly elevated NAF% values (e.g., ≥0.90) produced a positive logit corresponding to an estimated probability of OSA greater than 50%.

As NAF% increased, the probability of OSA rose exponentially.

The association between NAF% and OSA severity was particularly evident among males and children with a BMI above the 99th percentile.

However, the authors acknowledged that these conclusions were based on a relatively small sample and should be interpreted cautiously.

Integrating PSG with body composition analysis may prove valuable in specific clinical settings [139].

Body composition analysis provides crucial information regarding adipose tissue distribution in adults—particularly in the cervical, thoracic, and visceral regions [140,141]—where excessive fat accumulation can critically impair upper airway patency.

Consequently, this integrated diagnostic approach could be especially advantageous in children with severe obesity and overt symptoms of SDB.

Moreover, it facilitates longitudinal monitoring of weight reduction interventions and their impact on OSA severity, thereby supporting more individualised therapeutic strategies.

Despite growing interest in identifying predictive markers of paediatric OSA based on body composition, a universally validated predictive formula has yet to be established. Most existing tools use combinations of anthropometric, demographic, and clinical variables—often in the form of risk scores or regression models.

However, findings have remained inconsistent across different populations, and none have demonstrated sufficient reliability to replace PSG in children.

Nevertheless, body composition analysis remains an important adjunctive tool in the clinical evaluation of paediatric OSA.

**Figure 5:**
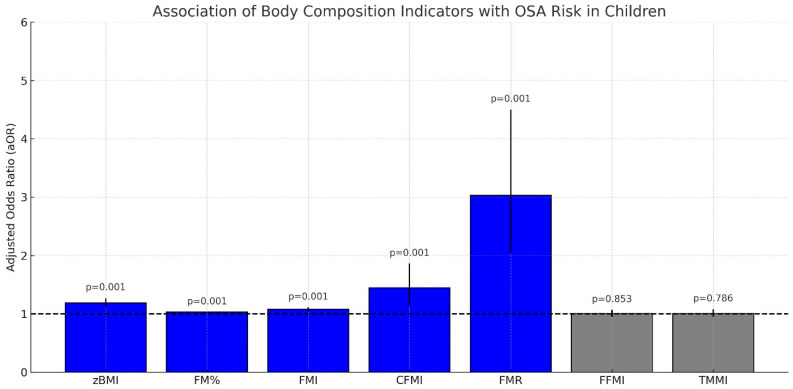
Adjusted odds ratios (aORs) and 95% confidence intervals (CIs) for body composition indicators associated with paediatric OSA risk. Indicators: FMR = fat-to-muscle mass ratio; zBMI = BMI z-score; FMI = fat mass index; CFMI = central fat mass index; FM% = fat mass percentage. The dashed vertical line at aOR = 1.0 represents no association. Statistically significant predictors are shown in blue; non-significant ones are shown in grey [45].

Among the available indices, the FMR has shown a strong association with OSA risk, underscoring the role of adiposity distribution in the development and severity of OSA in children and adolescents Figure 5 [45].

The indicators significantly associated with increased OSA risk include zBMI, BF%, FM index (FMI), central fat mass index (CFMI), and FMR.

FMR demonstrated the highest adjusted odds ratio (aOR), indicating the strongest correlation with OSA risk

Table 1 summarises the principal anthropometric indices—BMI, NC, NHR, NWR, and abdominal circumference—and the available evidence regarding their association with OSA.

The reported thresholds, derived from recent studies, provide practical guidance for OSA risk stratification, although they should be integrated with clinical and instrumental evaluations.

In children under 7, otorhinolaryngological (ORL) comorbidities (e.g., ATH) predominate, whereas in older children, body composition becomes a more significant contributor to OSA risk.

PSG is considered the gold standard for diagnosis, enabling a comprehensive assessment of nocturnal respiratory events (Table 2).

Alternatively, overnight pulse oximetry—measured via the oxygen desaturation index (ODI)—has been proposed as an accessible screening tool, with values ≥ 7.9 events/hour strongly associated with severe OSA.

Body composition analysis techniques, such as DXA, BIA, and ADP, allow for risk stratification based on fat distribution, particularly relevant in obese children.

Additional tools, including the Mallampati classification, end-tidal CO_2_ monitoring, and video recordings, further enrich the diagnostic protocol in centres with greater clinical complexity (Table 2).

The diagram (Table 3) provides clinicians with a guide for the initial evaluation of suspected paediatric OSA, integrating clinical history, anthropometric measurements, and instrumental screening tools.

Early identification of critical thresholds for BMI, NHR, NC>, and ODI facilitates prioritisation of access to PSG.

Body composition assessment using DXA, BIA, or ADP further supports an individualised risk stratification approach.

The pathway culminates in an integrated multidisciplinary strategy, essential for the comprehensive management of paediatric patients.

Key challenges and future perspectives. 

Clinical examination alone exhibits low sensitivity; however, the addition of simple measurements, such as the NHR or the NWR, enhances the ability to identify obese children at increased risk of OSA.

PSG remains essential for diagnostic confirmation and severity quantification, but ODI and targeted anthropometric assessments contribute to optimising waiting list management.

Fat distribution is critical rather than total adiposity; DXA, BIA, or ADP should be integrated into the diagnostic pathway.

Adopting practical thresholds (BMI ≥ 29 kg/m^2^, NC 35–36 cm, NHR > 0.23–0.25) enables evidence-based triage, drawing from validated studies.

The clinical application of body composition analysis presents several challenges, including variability in measurement methodologies, difficulties in data interpretation, and inconsistent correlations with clinical outcomes.

Standard techniques such as BIA [143] and DXA accurately estimate FM and FFM.

However, these methodologies often fail to precisely assess fat distribution in anatomically critical regions, such as the pharyngeal and cervical areas [58,138].

Ethnicity- and sex-specific predictive equations used to estimate FFM from BIA data allow for more accurate assessments of ethnic differences in FFM and FM in children.

In contrast, generic equations may obscure such distinctions [143].

A preliminary study conducted in 20 children (mean age: 14.8 ± 0.6 years for AHI < 1.5 vs. 14.9 ± 0.7 years for AHI ≥ 1.5; *p* = 0.94) reported a strong correlation between AHI and visceral adipose tissue measured by DXA (*r* = 0.67; *p* < 0.01) [144].

A significant limitation is the lack of normative reference values specific to age and sex for the paediatric population.

Integrating body composition data with functional assessments of upper airway dynamics remains challenging, often requiring complementary investigations such as PSG or MRI.

Therefore, although body composition analysis provides valuable insights, it must always be interpreted within the broader context of clinical evaluation to guide accurate diagnosis and effective therapeutic planning. No universally accepted composite scoring system integrating anthropometric and body composition parameters for paediatric OSA risk stratification exists. While models combining NHR, zBMI, and NWR have shown promise individually or in pairs, their integration into a single, validated predictive tool remains a key research priority. The next generation of predictive models should incorporate variables reflecting upper airway fat distribution (e.g., NAF%), anthropometric ratios (e.g., NHR, NWR), and metabolic indicators (e.g., presence of metabolic syndrome components), ideally supported by multicentre prospective validation. A critical limitation in current research is the lack of universally accepted normative data for these parameters across paediatric age groups, sexes, pubertal stages, and ethnic backgrounds. This variability complicates risk stratification and the development of reliable predictive tools. Future studies should adopt large-scale, multicentre designs that adequately represent diverse populations and age groups, standardised measurement protocols, and longitudinal follow-up. Such frameworks would establish robust normative reference ranges and facilitate the creation of clinically applicable, age- and sex-specific risk models.

Furthermore, available evidence suggests that targeted interventions, such as weight-loss programmes and adenotonsillectomy, can induce significant improvements in anthropometric measures (e.g., reductions in zBMI, NC, and fat distribution indices) and contribute to the partial or complete resolution of OSA. For example, weight loss has been associated with decreased pharyngeal fat volume and lower AHI values, while adenotonsillectomy has demonstrated efficacy in reducing upper airway obstruction and improving OSA severity in children, particularly those with ATH. Future studies should systematically assess these longitudinal changes using standardised anthropometric and compositional metrics to inform personalised treatment strategies and follow-up protocols.

While not yet routinely implemented in clinical practice, several emerging diagnostic methods show considerable potential to enhance predictive accuracy for OSA, particularly in obese children [145,146].

US elastography enables the evaluation of soft tissue stiffness, such as the pharyngeal walls and tongue, offering biomechanical insights into upper airway collapsibility.

Assessment of respiratory muscle strength through measurements of maximal inspiratory and expiratory pressures provides valuable information on neuromuscular competence, as reduced muscle strength may increase the risk of airway instability during sleep [147,148].

Furthermore, three-dimensional craniofacial imaging and upper airway MRI allow for detailed, non-invasive morphological analysis, facilitating the identification of anatomical risk factors—such as retrognathia and mandibular hypoplasia—that may contribute to upper airway obstruction [149,150,151], also in obese children.

Emerging evidence highlights that cardiovascular risk in paediatric patients is influenced not only by the presence of obesity, but also by incremental increases in specific anthropometric indices, such as WC and WHR [152,153].

These parameters are independently associated with metabolic dysfunction and serve as reliable indicators of central adiposity. In particular, a recent study [154] demonstrated that even modest increases in WHR may significantly elevate cardiovascular risk, regardless of BMI status. This aligns with our aim to identify clinically accessible and physiologically meaningful predictors that reflect fat distribution and associated health outcomes in children with suspected OSA.

**Figure 6:**
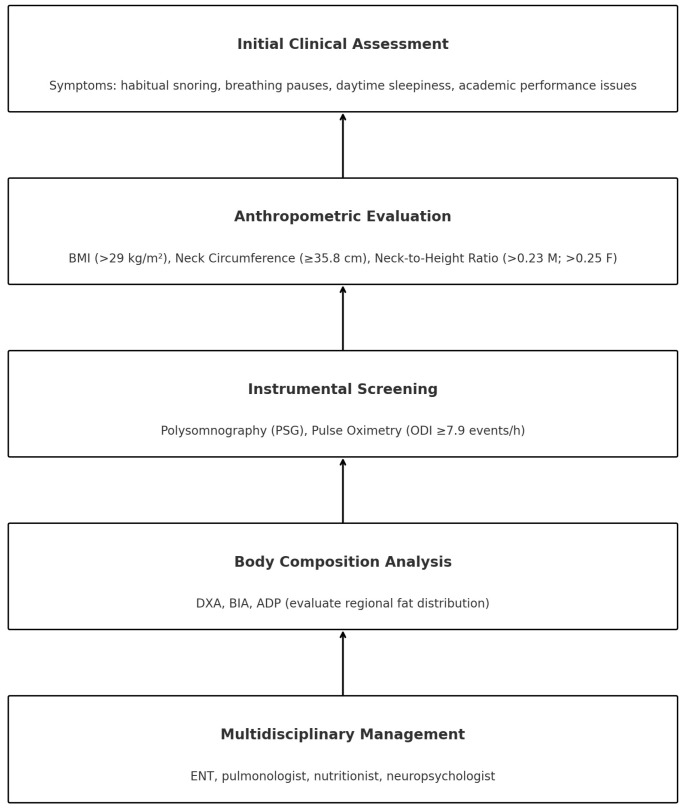
Flow-chart of a five-step clinical pathway for stratification and integrated management of paediatric OSA in children with obesity: (1) initial clinical assessment, (2) anthropometric evaluation (BMI, NC, NHR), (3) instrumental screening (overnight oximetry or PSG if ODI ≥ 7.9 h^−1^), (4) body composition analysis (DXA, BIA, ADP), and (5) multidisciplinary management (ENT specialist, pulmonologist, nutritionist, psychologist). Abbreviations: ADP, air-displacement plethysmography; BIA, bioelectrical impedance analysis; BMI, body mass index; DXA, dual-energy X-ray absorptiometry; ENT, ear–nose–throat specialist; NC, neck circumference; NHR, neck-to-height ratio; ODI, oxygen desaturation index; OSA, obstructive sleep apnoea; PSG, polysomnography.

The flow chart (Figure 6) outlines a stepwise approach starting from the initial clinical assessment, focusing on key symptoms such as habitual snoring, breathing pauses, daytime sleepiness, and academic issues. Anthropometric evaluation includes BMI, NC, and NHR, with sex-specific thresholds. Instrumental screening relies on PSG and pulse oximetry (ODI ≥ 7.9 events/h). Body composition analysis incorporates techniques such as DXA, BIA, and ADP to assess regional fat distribution. Finally, multidisciplinary management involves referral to specialists (ENT, pulmonologist, nutritionist, neuropsychologist) for integrated care.

## 6. Conclusions

In obese children with suspected OSA, evaluated through PSG and body composition assessment, specific anthropometric and compositional indicators—such as the NHR, NWR, FMR, and NAF%—show significant associations with OSA severity Table 4.

Clinical implications: These parameters may assist in identifying high-risk children and prioritising access to diagnostic testing.

Clinicians should consider including NHR and body composition measures in standard assessments, particularly in settings with limited PSG availability.

Future research should prioritise multicentre, prospective studies that integrate multimodal data—including clinical, anthropometric, body composition, and imaging parameters—to enhance risk stratification and personalised care. Technological advances, such as three-dimensional imaging, MRI, ultrasound elastography, and wearable diagnostic devices, offer promising avenues to refine paediatric OSA assessment and monitor disease progression. Moreover, strategies should be developed to link early body composition metrics with long-term cardiometabolic and neurocognitive outcomes, using longitudinal designs and machine-learning approaches to overcome current predictive limitations.

## Figures and Tables

**Table 1:** Anthropometric parameters and otorhinolaryngological comorbidities associated with paediatric obstructive sleep apnoea. The table lists body-mass-related indices (BMI, zBMI), cervical measures (NC, NHR), central adiposity markers (NWR, waist circumference) and upper-airway conditions (adenotonsillar hypertrophy, Mallampati grade). For each variable, we report (i) the key supporting evidence, (ii) clinically useful thresholds, and (iii) sensitivity/specificity or effect size when available.

Parameter	Main Evidence	Useful Thresholds/Associations
BMI	A cut-off of 29.2 kg/m² predicts severe OSA (sensitivity 81%, specificity 49%) [99]	Use as an alert, but not sufficient on its own
NC	Average increase of +1 cm in the OSA group vs. controls [101]	NC ≥ 35.8 cm predicts OSA severe [99]
NHR	NHR > 0.25 (all) OR 3.47 for AHI > 2 [97]	>0.23 males, >0.25 females per oAHI > 5 [98]
NWR	RR 1.97 for an increase of 0.1 units in the NWR; predictive, especially in the obese [142]	Significant association only in overweight/obese children
Waist and hip circumferences	Waist +3 cm and hips +4 cm in OSA compared to non-OSA [101]	Waist ≥93.5 cm associated with severe OSA [99]
Comorbidities ORL	Relevant ATH in children <7 years of age; obesity prevails in the older ones [100]	Evaluate tonsillar size and Mallampati score as additional factors

Abbreviations: AHI, apnoea–hypopnoea index; ATH, adenotonsillar hypertrophy; BMI, body mass index; NC, neck circumference; NHR, neck-to-height ratio; NWR, neck-to-waist ratio; OSA, obstructive sleep apnoea; OR, odds ratio; RR, relative risk; sens, sensitivity; spec, specificity.

**Table 2:** Instrumental assessment tools for paediatric obstructive sleep apnoea and their main strengths. The table summarises overnight polysomnography (PSG), the oxygen desaturation index (ODI), pulse oximetry, body composition techniques (dual-energy X-ray absorptiometry [DXA], bioelectrical impedance analysis [BIA], air-displacement plethysmography [ADP]), and ancillary evaluations (Mallampati scoring, end-tidal CO_2_ monitoring, video recording). We report the principal outcome measure, clinical advantages, and key reference thresholds for each tool.

Instrument	Strengths
PSG	Gold standard: defines AHI/oAHI, desaturations, and hypoventilation
ODI	Rapid screening if PSG not available; ODI ≥ 7.9 events/h: OR 17.2 for severe OSA [99]
DXA	Quantify regional fat; NAF ratio % associated with oAHI in males with BMI > 99th centile [138]
Multi-frequency BIA	Evaluate FM%, FMI, and muscle mass; high FM (not lean body mass) correlates with OSA risk [45]
ADP (BOD-POD^®^ Paediatric option)	Validated technique 2–6 years for body composition follow-up [135]
Other measures	Mallampati, end-tidal CO_2_, video recording included in advanced PSG protocols [138]

Abbreviations: ADP, air-displacement plethysmography; AHI, apnoea–hypopnoea index; BIA, bioelectrical impedance analysis; BMI, body mass index; DXA, dual-energy X-ray absorptiometry; FM, fat mass; FMI, fat mass index; ODI, oxygen desaturation index; oAHI, obstructive AHI; OSA, obstructive sleep apnoea; PSG, polysomnography.

**Table 3:** Stepwise triage algorithm for the assessment and management of paediatric obstructive sleep apnoea (OSA) in obesity. The flow chart integrates six sequential steps: (i) clinical history and symptom screening, (ii) anthropometric thresholds (BMI z-score, neck circumference, neck-to-height ratio), (iii) overnight oximetry/polysomnography criteria, (iv) ear–nose–throat evaluation, (v) body composition techniques (DXA, BIA, ADP), and (vi) referral to weight management or surgical pathways, with recommended follow-up intervals for each risk stratum.

Clinical Pathway Stage	Activity/Exam	Benchmarks or Decision Thresholds
Anamnesis	Habitual snoring (≥3 nights/week), breathing pauses, daytime sleepiness, and academic performance	The presence of ≥2 reported symptoms suggests an increased risk of OSA and indicates the need for further instrumental screening (ODI or PSG)
Standardised anthropometry	Weight, height, NC, WC; NHR and NWR calculation. PSG priority if ≥ any of the following:	BMI > 29 kg/m^2^ at >+2.5 D NHR > 0.23 (M)/0.25 (F) NC ≥ 35.8 cm or high NWR
Instrumental screening	Nocturnal pulse oximetry: if ODI ≥ 7.9 events/h	→PSG Direct
Body composition	DXA (or BIA if DXA is not available), ADP	Specialist centres to assess fat distribution. ADP is helpful in the <6 years for nutritional follow-up
Multidisciplinary management	ENT, pulmonologist, nutritionist and, if cognitive deficits are present, neuropsychologist	Confirmed or severe OSA (oAHI > 5), ATH, neurocognitive or behavioural disorders, need for therapeutic-nutritional planning

Abbreviations: ADP, air-displacement plethysmography; ATH, adenotonsillar hypertrophy; BIA, bioelectrical impedance analysis; BMI, body mass index; DXA, dual-energy X-ray absorptiometry; ENT, ear–nose–throat; NHR, neck-to-height ratio; NWR, neck-to-waist ratio; OSA, obstructive sleep apnoea; PSG, polysomnography; WC, waist circumference.

**Table 4:** Validated anthropometric and body composition predictors of paediatric OSA (n = 152): operational definitions, sex-specific cut-offs, strengths, and methodological limitations.

Parameter	Definition	Strengths	Limitations	Thresholds/Sex-Specific Notes
NHR	NC/height	Simple; associated with AHI	May vary by growth stage	>0.23 males; >0.25 females (AHI > 5)
NWR	Neck/waist circumference	Indicates fat distribution	Thresholds less standardised	↑ Risk with NWR > 0.43
WHR	Waist/hip circumference	Estimate visceral obesity	Less accurate in children	Less consistent in paediatrics
FMR	FM/muscle mass	Correlation strongly with OSA	Requires body composition tools	Strongest predictor among BIA metrics
NAF%	% neck fat/abdominal fat	Reflects upper airway fat burden	Based on DXA or MRI	>0.90 = ↑ OSA risk (males, BMI > 99°)

Abbreviations: AHI, apnoea–hypopnoea index; BIA, bioelectrical impedance analysis; DXA, dual-energy X-ray absorptiometry; FM, fat mass; FMI, fat–mass index; FMR, fat-to-muscle mass ratio; NAF %, neck-to-abdominal fat percentage; NHR, neck-to-height ratio; NWR, neck-to-waist ratio; WHR, waist-to-hip ratio.

## Data Availability

Data were derived from public domain resources.
